# Do circulating sphingolipid species correlate with age? A study in a normoglycemic biracial population

**DOI:** 10.1007/s10522-025-10244-9

**Published:** 2025-05-05

**Authors:** Naser Aliye Feto, Peace Asuzu, Jim Wan, Frankie Stentz, Sam Dagogo-Jack, Nawajes Mandal

**Affiliations:** 1 Departments of Ophthalmology, Anatomy and Neurobiology, University of Tennessee Health Science Center, 930 Madison Ave., Memphis, TN 38163 USA; 2 Division of Endocrinology, Diabetes and Metabolism, Department of Medicine, University of Tennessee Health Science Center, Memphis, TN 38163 USA; 3 Department of Preventive Medicine, University of Tennessee Health Science Center, Memphis, TN 38163 USA; 4 General Clinical Research Center, University of Tennessee Health Science Center, Memphis, TN 38163 USA; 5 Research, Memphis VA Medical Center, Memphis, TN 38104 USA

**Keywords:** Sphingolipid, Aging, Ceramide, Glycosphingolipid, Sphingomyelins, Sphingosine

## Abstract

Sphingolipids (SPLs) are essential membrane lipids with significant bioactive roles involved in various cellular processes, and their alterations have been found to be linked to many diseases, including age-related diseases. However, comprehensive studies on the association of plasma sphingolipids with aging in large, diverse cohorts remain limited. The objective of this study was to investigate the relationship between plasma sphingolipid levels and aging in a cohort of 240 normoglycemic, biracial individuals (Black and White), aged 19–65 years. Using a targeted lipidomics approach, we measured 76 sphingolipid species using liquid chromatography-tandem mass spectrometry (LC–MS/MS) in picomole/mL and determined changes in their levels with age and their correlations with aging. We found significant age-related changes in several sphingolipid species, including ceramide C18:1 and several very long-chain sphingomyelins (VLC SMs), such as C28:1 and C30:1, increases with age, showing a positive correlation. On the other hand, glycosphingolipids (monohexosylceramide, MHC; lactosylceramide, LacCer) and sphingosine (So) showed strong negative correlations with aging. A significant correlation was also observed between the ratios of saturate/monosaturated sphingolipid species with aging. In conclusion, our findings provide novel insights into the dynamic changes of circulating sphingolipids with aging. Specific sphingolipid species, such as Ceramide C18:1 and SM, accumulate with age, while others, including MHC, LacCer, and So decrease. These results suggest that the plasma SPL profile may provide valuable information about healthy aging and age-associated disease conditions.

**Supplementary Information:**

The online version contains supplementary material available at 10.1007/s10522-025-10244-9.

## Introduction

Sphingolipids (SPLs) are vital components of eukaryotic cells. They play critical roles as structural components of cellular membranes, besides their fundamental roles as bioactive lipids in cellular signaling, inflammation, regulation of cell growth, and apoptosis (Bartke and Hannun 2009; Hannun and Obeid 2018; Quinville et al. 2021). Sphingolipid metabolism is convolutedly linked with several physiological and pathological processes, and their dysregulation has been implicated in different age-related metabolic disorders, including type 2 diabetes, cardiovascular diseases, neurodegenerative disorders, and cancer (Czubowicz et al. 2019; Mandal et al. 2024; Owei et al. 2016; Shu et al. 2022; Trayssac et al. 2018).

Aging is characterized by the progressive decline of physiological functions with time, fundamentally accompanied by alterations in cellular signaling, metabolism, and failure to sustain homeostasis, thereby increasing exposure to several neurological and metabolic diseases (Czubowicz et al. 2019; Kirkwood 2005; Li and Kim 2021; St Sauver et al. 2023). Several bodies of research in both animal models and humans have demonstrated that sphingolipid metabolism undergoes significant alterations during aging and may contribute to the onset of age-related diseases (Argraves et al. 2011; Bozek et al. 2017; Cutler et al. 2014; Huang et al. 2012, 2014; Jove et al. 2013; Lewis et al. 2018; Liu et al. 2013). In humans, age-related changes in sphingolipids have been reported in different tissues, including the brain and cardiovascular system, senescent cells, and age-associated diseases like dementia (Czubowicz et al. 2019; McGrath et al. 2020; Trayssac et al. 2018). For instance, elevated ceramide levels in the plasma and tissues of elderly individuals are associated with increased risk for age-related diseases such as Alzheimer’s disease and cardiovascular conditions (Czubowicz et al. 2019; Obas and Vasan 2018; Wessells and Bodmer 2007).

Though from many mechanistic-based cell culture studies, model organisms, and human studies in the recent past, perturbations in sphingolipid metabolism are associated with the development and progression of many age-related diseases, their longitudinal association with age at the population level and how these lipid levels change, especially for the general healthy population, have not been investigated. Current knowledge on sphingolipid-age association in humans is generated from either smaller cohorts, narrower age gaps between the participants, limited to one race, and sometimes with only a limited number of SPL species (Collino et al. 2013; Darst et al. 2019; Gonzalez-Covarrubias et al. 2013; Jove et al. 2017; Mielke et al. 2015; Montoliu et al. 2014; Pradas et al. 2022). We report the association of 76 plasma sphingolipid species measured by targeted mass spectrometric analysis with age from 240 healthy human participants from Black and White races who were metabolically normoglycemic and aged between 19 and 65 years.

## Materials and methods

### Participants in the study

We recruited 240 participants, stratified into five age groups: 19–29, 30–39, 40–49, 50–59, and 60–65 years. Inclusion criteria required normal fasting plasma glucose (< 100 mg/dL) and/or normal glucose tolerance (2 h plasma glucose < 140 mg/dL), as confirmed by a 75 g oral glucose tolerance test. These criteria were adapted from the previously established POP-ABC study protocol with some modifications (Dagogo-Jack et al. 2013, 2014, 2011). Exclusion criteria included the use of medications affecting glucose or body weight, as well as participation in weight-loss interventions. The study participants underwent study procedures at the University of Tennessee Health Science Center (UTHSC), General Clinical Research Center (GCRC) after providing written informed consent to participate voluntarily. The study adhered to the ethical principles of the Declaration of Helsinki and was approved by the University of Tennessee’s Institutional Review Board.

### Examinations

Participants fasted overnight prior to undergoing study procedures at the UTHSC General Clinical Research Center (GCRC). During the baseline visit, a comprehensive assessment of medical history and physical parameters was conducted. Measurements included height, weight, waist circumference, and fasting plasma glucose, with blood samples collected for lipidomics analysis and subsequently stored at − 80 °C (Dagogo-Jack et al. 2013, 2014, 2011). Body mass index (BMI) was calculated by dividing weight (kg) by height (m^2^). Fasting plasma glucose was determined using the YSI glucose analyzer (Yellow Spring Instruments Co., Inc., Yellow Spring, OH).

## Lipidomics

### Sphingolipid extraction and analysis

Sphingolipid composition was analyzed using 40 µL of frozen plasma from each subject, following a published protocol (Mandal et al. 2024; Paranjpe et al. 2019) at the Lipidomic Core, Virginia Commonwealth University. Internal standards used for the analysis were purchased from Avanti Polar Lipids (Alabaster, AL). Internal standards were added to samples in 10 µL ethanol:methanol:water (7:2:1) as a cocktail of 250 pmol each. Standards for sphingoid bases and sphingoid base 1-phosphates were 17-carbon chain length analogs: C17-sphingosine, (2S,3R,4E)-2-aminoheptadec-4-ene-1,3-diol (d17:1-So); C17-sphinganine, (2S,3R)-2-aminoheptadecane-1,3-diol (d17:0-Sa); C17-sphingosine 1-phosphate, heptadecasphing-4-enine-1-phosphate (d17:1-So1P); and C17-sphinganine 1-phosphate, heptadecasphinganine-1-phosphate (d17:0-Sa1P). Standards for N-acyl sphingolipids were C12-fatty acid analogs: C12-Cer, N-(dodecanoyl)-sphing-4-enine (d18:1/C12:0); C12-sphingomyelin, N-(dodecanoyl)-sphing-4-enine-1-phosphocholine (d18:1/C12:0-SM); and C12-glucosylceramide, N-(dodecanoyl)-1-β-glucosyl-sphing-4-eine (d18:1/C12:0-MHC). The samples were mixed with MeOH:CHCl₃ (2:1), sonicated for 2 min, and incubated at 48 °C for 6 h. After centrifugation at 5000 rpm for 20 min, the supernatant was transferred, vacuum-dried, and reconstituted in 500 µL methanol. The final solution was centrifuged again and transferred to injection vials for high performance liquid chromatography (HPLC) separation and mass spectrometric analysis.

### Liquid chromatography-mass spectrometry (LC–MS)-based analysis of sphingolipids

Liquid chromatography-mass spectrometry (LC–MS) analysis of sphingolipids was performed as described previously (Mandal et al. 2024). Sphingolipids were separated using HPLC (Shimadzu Nexera X2 LC-30AD) with a SIL-30AC auto-injector and DGU-20A5R degassing unit, following established protocols (Mandal et al. 2024). The ABI Sciex Triple Quad 5500 Mass Spectrometer was used for sphingolipid identification and quantification via a targeted assay (Shaner et al. 2009). Q1 and Q3 were set to select specific precursor and product ion pairs unique to sphingolipids, with N2 gas facilitating collision-induced dissociation in Q2. Multiple-reaction monitoring in positive-ion mode was employed for analysis. Quantification was based on elution profiles for each MRM (multiple reaction monitoring) pair, and internal standards were used to calculate sphingolipid species concentrations. Mass spectrometer parameters included Curtain Gas (30 psi), CAD (collisionally-activated dissociation, Medium), Ion Spray Voltage (5500 V), and others, as detailed in previous reports (Mandal et al. 2024; Qi et al. 2017; Shaner et al. 2009; Wijesinghe et al. 2010; Wilmott et al. 2019). We have determined the sphingolipids at the ‘molecular species level’ using the established procedure of Dr. Al Merrill in 2009, and it has been a standard for SPL analysis for years (Shaner et al. 2009). Using tandem mass spectrometry with two rounds of MS with optimized MRM transitions for quantitative measurement of complex sphingolipids, we obtained the total mass (precursor Q1 m/z) and the second round of MS fragments of the molecule (product Q3 m/z). Fragmentation of most complex sphingolipids involves dehydration at the three position, dehydration/loss of the headgroup from the one position, and cleavage of the N-acyl fatty acid to a conjugated carbocation of m/z 264.4 for d18:1-based species. Through targeted analysis, we selected sphingolipid species only with the backbone of d18:0 sphingosine (m/z 264.4). We, therefore, simplified our nomenclature by providing the information only for the esterified N-linked fatty acids, which are the second part of the LIPID MAPS nomenclature (Liebisch et al. 2020). For example, ceramide d18:1/C18:0 as Cer C18:0, glucosyl or galactosyl HexCer d18:1/C18:1 as MHC (monohexosylceramide) C18:1, and sphingomyelin d18:1/C24:0 as SM C24:0. All the lipids and their molecular species composition and their LIPID MAPS nomenclature are included in Supplementary Table (Table S1).

### Statistical analysis

Data are presented as means ± standard deviation (SD). Differences between groups for continuous and categorical variables were assessed using analysis of variance (ANOVA) and the chi-square test for categorical variables. Adjusted analyses were conducted using linear regression models. Correlations were employed to examine the association between age and sphingolipid species. Statistical significance was defined as P < 0.05 (two-tailed). All statistical analyses were conducted using SAS 9.4 software (SAS Institute Inc., Cary, NC) and GraphPad Prism Version 8.4.3 (471) (GraphPad Software, LLC, Boston, MA).

## Results

### Baseline characteristics

The baseline characteristics of study subjects comprising the five age groups are as in Table 1. The mean values of the baseline show that there was a fair representation of the groups except the last group (60–65 y), which comprises a total participation of 15 (n = 15) (Table 1). The group showed no significant variation at baseline in weight, BMI, and waist circumference. However, the values of FPG and 2hPG were higher in the relatively older group than the younger group. Otherwise, all in all, it was a relatively fair starting point.

**Table 1:** Baseline characteristics of the study participants

Characteristics	All	19–29y	30–39y	40–49y	50–59y	60–65y	*p* value
Number	240	46	47	64	68	15	
Black/White	129/110	25/21	30/17	37/27	31/37	6/9	0.26
Female/Male	149/90	28/18	25/22	39/25	46/22	12/3	0.33
FPG (mg/dl)	91.4 ± 6.9	87.5 ± 6.0	90.6 ± 6.5	92.9 ± 6.9	92.4 ± 6.4	94.5 ± 8.9	0.0001
2hPG (mg/dl)	122.8 ± 26.3	111.4 ± 16.9	120.0 ± 23.4	123.1 ± 28.5	130.1 ± 29.1	132.5 ± 25.5	0.002
Weight (kg)	85.1 ± 19.5	82.7 ± 23.5	91.6 ± 21.7	86.2 ± 15.8	81.4 ± 8.0	85.8 ± 15.3	0.07
BMI (kg/M^2^)	29.4 ± 6.2	27.8 ± 6.7	30.7 ± 6.7	29.7 ± 5.5	29.0 ± 6.3	31.6 ± 4.8	0.10
Waist (cm)	93.4 ± 15.0	88.1 ± 17.1	96.4 ± 15.1	95.4 ± 11.8	92.9 ± 16.0	95.7 ± 12.6	0.06

Data are shown as mean ± SD. 2hrPG, 2 h plasma glucose during 75 g oral glucose tolerance test (to convert the values for glucose to millimoles per liter, multiply by 0.0555); *BMI* body mass index, and *Waist* waist circumference

### Distribution of the major classes of plasma sphingolipids across different age groups

**Fig. 1:**
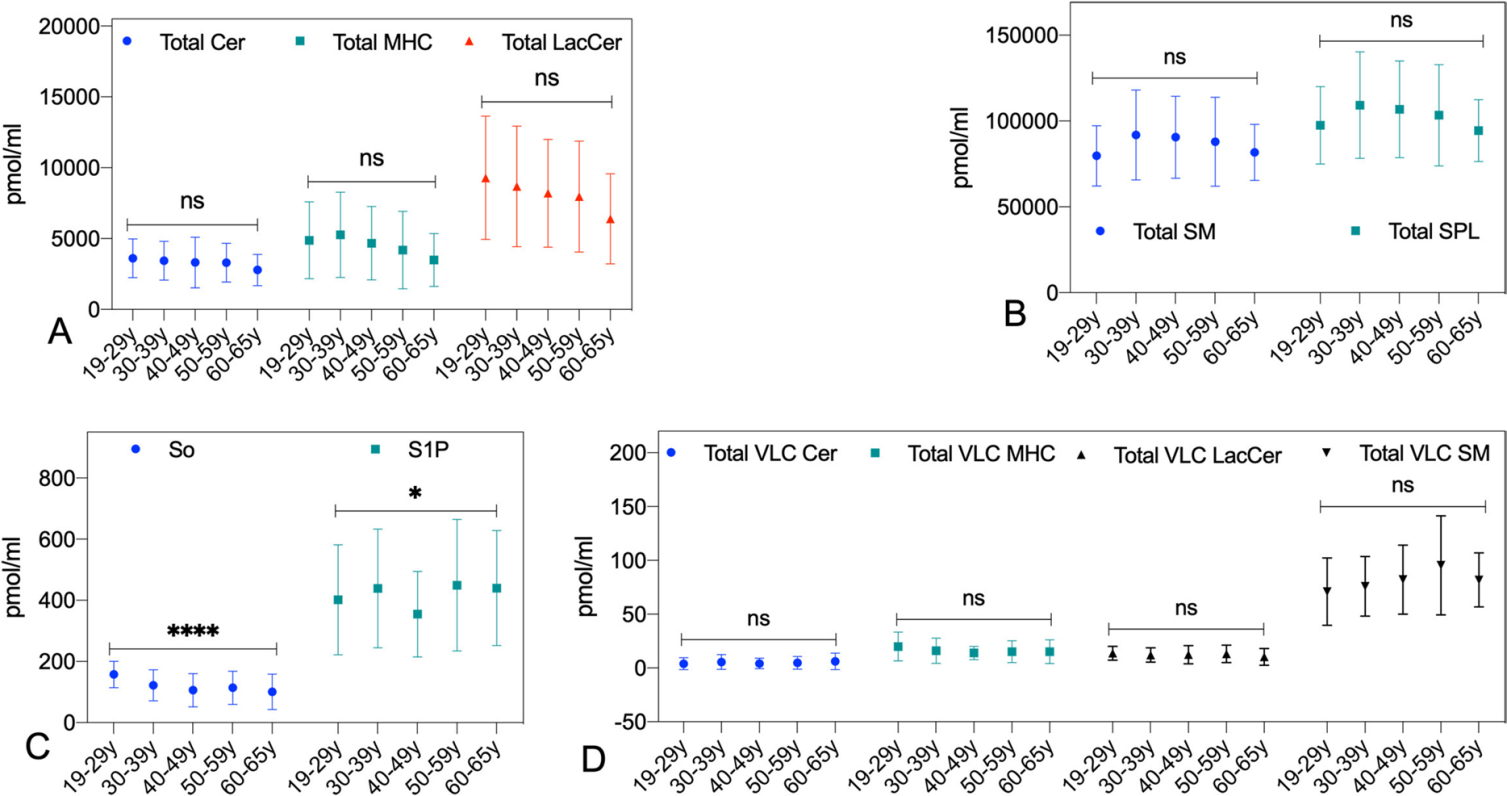
The changes in levels of sphingolipid classes across different age groups. **A** Total ceramide (Cer), total monohexosylceramide (MHC), total lactosylceramide (LacCer), **B** Total sphingomyelin (SM), total sphingolipids (SPL), **C** Total VLC ceramide, total VLC monohexosylceramide, total VLC lactosylceramide, Total VLC sphingomyelin, **D** Sphingosine (So) and Sphingosine 1-phosphate (S1P)

We determined the plasma levels of 76 sphingolipid species comprising ceramide (Cer), monohexosylceramide (MHC), lactosylceramide (LacCer), sphingomyelin (SM), sphingosine (So), dihydrosphingosine (DHSo), sphingosine 1-phosphate (S1P), and dihydrosphingosine 1-phosphate (DH-S1P) from plasma samples of 240 individuals across the five age groups. First, we determined if there was any difference in the pmol/mL levels of these classes of SPL. As shown in Fig. 1A and B, no significant difference was observed in the total Cer, MHC, LacCer, SM, and the total SPL. However, we found that some trends in total MHC and LacCer levels decrease with age, and the total SM and total SPL increases (Fig. 1A and B). We observed a significant age-related variation in the plasma levels of sphingosine (So) (p < 0.0001) and sphingosine-1-phosphate (S1P) (p = 0.02), with a marked decline in So levels as individuals aged (Fig. 1C). In contrast, the plasma concentrations of dihydrosphingosine (DHSo) and dihydrosphingosine-1-phosphate (DHS1P) remained largely unchanged across the age groups.

We then looked at the total VLC SPL species, and no significant difference was observed in their levels except for some increasing and decreasing trends in different groups (Fig. 1D). The error bars in Fig. 1 indicate significant variability, reflecting the heterogeneity in sphingolipid levels across individuals; however, the data suggest subtle but notable trends in sphingolipid profiles with aging.

### Relative composition of major classes of plasma sphingolipids across different age groups

**Fig. 2:**
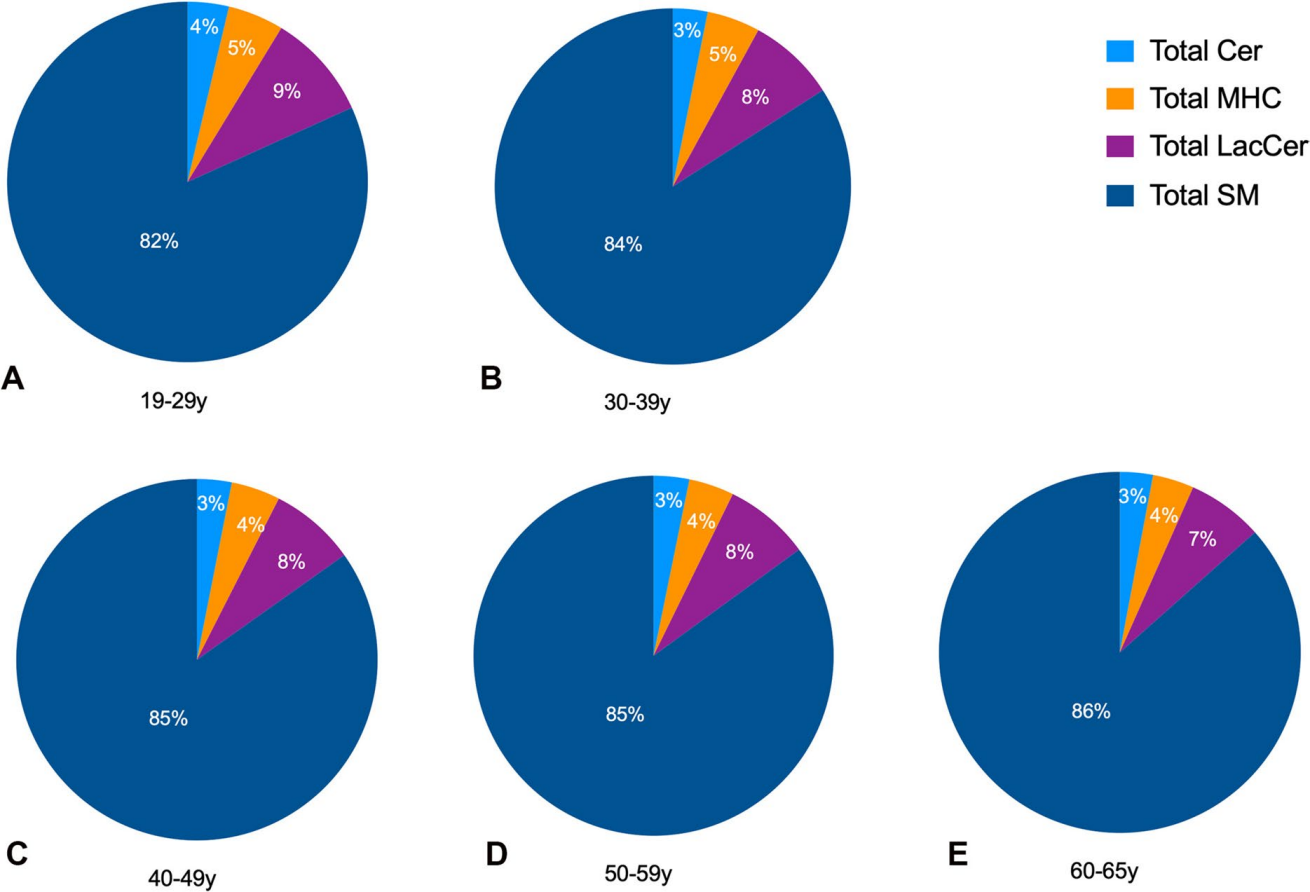
The relative proportion of total ceramide (Cer), total monohexosylceramide (MHC), total lactosylceramide (LacCer) and total sphingomyelin (SM) in different age groups: **A** 19–29 y, **B** 30–39 y, **C** 40–49 y, **D** 50–59 y, and **E** 60–65 y

We then investigated the molar composition or the relative expression of these four major classes of SPL across the ages- Cer, MHC, LacCer, and SM. Our study reveals that plasma sphingolipid composition remains largely stable across age groups, with only subtle changes observed (Figs. 2A–E). The proportions of Cer, MHC, LacCer, and SM showed minimal variation, remaining within narrow ranges (3–4%, 4–5%, 7–9%, and 82–86%, respectively) throughout aging (Figs. 2A–E). Interestingly, though, we noticed a gradual increase in the levels of SM % from 82% in the 19–29 age group to 86% in the 60–65 age group, suggesting a slight shift in sphingolipid distribution towards increasing sphingomyelin with advancing age (Figs. 2A–E). These results may imply that while overall sphingolipid profiles do not drastically change with age, there may be a subtle, age-related accumulation of sphingomyelin in plasma.

### Differentially expressed individual SPL species across different age groups

Though we did not observe the total SPL or the total of the major classes of SPL differing significantly among the age groups, we found that some of the individual species among those classes differed. Among the Cer species, we observed that the level of Cer C18:1 was elevated in conjunction with age but lost significance when adjusted for BMI, FPG, sex, and ethnicity (Table 2). Accordingly, we noticed the ratio of Cer C18:0/C18:1 decreased consistently with increases in age and remained significant after adjusting for covariates (from 11.6 ± 7.1 in 19–29 y to 6.6 ± 3.9 in 60–65 y group) (p = 0.003) (Table 2).

**Table 2:** Comparison of plasma SPL species levels between different age groups

SPL	All	19–29y	30–39y	40–49y	50–59y	60–65y	*p* value^b^	*p* value^c^
Cer								
C18:1	6.0 ± 3.5	4.6 ± 2.7	6.1 ± 4.2	6.5 ± 2.9	6.3 ± 3.8	6.9 ± 3.1	0.02	0.14
C18/C18:1	8.8 ± 5.7	11.6 ± 7.1	9.9 ± 6.5	7.5 ± 5	7.9 ± 4.1	6.6 ± 3.9	0.0004	0.003
MHC								
C18:1	59.2 ± 49.6	42.1 ± 41.4	61.4 ± 44.1	72.8 ± 50.3	58.6 ± 56.8	51.9 ± 39.0	0.02	0.02
C26:0	7.8 ± 5.6	9.7 ± 5.9	8.0 ± 5.9	7.6 ± 5.8	7.0 ± 5.2	5.2 ± 2.1	0.03	0.32
C28:0	1.4 ± 1.4	2.2 ± 1.7	1.4 ± 1.5	1.1 ± 1.0	1.3 ± 1.3	0.9 ± 0.6	0.0001	0.0009
C34:1	0.6 ± 0.5	0.8 ± 0.6	0.6 ± 0.5	0.5 ± 0.4	0.6 ± 0.5	0.5 ± 0.3	0.03	0.22
C34:0	0.3 ± 0.3	0.4 ± 0.3	0.3 ± 0.3	0.2 ± 0.2	0.3 ± 0.3	0.1 ± 0.1	0.001	0.01
C18/C18:1	1.2 ± 0.9	1.7 ± 1	1.2 ± 0.8	1 ± 0.9	1 ± 0.9	1.1 ± 1	0.001	0.0009
C26/C26:1	0.7 ± 0.4	0.9 ± 0.4	0.6 ± 0.3	0.6 ± 0.3	0.6 ± 0.4	0.6 ± 0.3	< 0.0001	0.002
LacCer								
C26:1	20.7 ± 16.4	25.6 ± 21.2	20.9 ± 17	20 ± 13.8	20.4 ± 15	9 ± 6.3	0.01	0.02
C28:1	1.9 ± 2	1.3 ± 0.7	1.4 ± 1.3	2.3 ± 3	2 ± 1.7	2.1 ± 1.8	0.03	0.04
C30:1	1.1 ± 1	0.7 ± 0.6	1 ± 0.7	1.2 ± 1.1	1.3 ± 1	1.2 ± 0.9	0.007	0.003
C32:0	2.2 ± 2.6	3.8 ± 2.8	2.9 ± 3	1.6 ± 2.2	1.5 ± 2	1.1 ± 1.2	< 0.0001	< 0.0001
C34:1	0.4 ± 0.8	0.07 ± 0.2	0.3 ± 0.6	0.43 ± 0.6	0.76 ± 1.2	0.4 ± 0.5	0.0004	0.0008
C34:0	0.3 ± 0.5	0.03 ± 0.1	0.3 ± 0.5	0.3 ± 0.3	0.5 ± 0.6	0.4 ± 0.6	< 0.0001	< 0.0001
C26/C26:1	0.7 ± 0.6	0.6 ± 0.4	0.6 ± 0.5	0.7 ± 0.7	0.6 ± 0.5	1.2 ± 1	0.01	0.02
SM								
C14:0	6271.3 ± 2145.2	5522.1 ± 1738.5	6382.4 ± 1785.8	5812.5 ± 2101.1	6987.2 ± 2418.6	6859.5 ± 2240.2	0.001	0.004
C18:1	6108.7 ± 2393.9	5167.23 ± 1507.75	6823.23 ± 2558.09	6524.65 ± 2367.86	6016.37 ± 2685.90	5580.6 ± 1865.0	0.006	0.01
C26:0	43.9 ± 19.8	38.2 ± 15.1	46.3 ± 21.9	45.9 ± 22.7	46.5 ± 18.9	35.3 ± 8.9	0.05	0.02
C28:1	18.5 ± 8.1	15.8 ± 8.1	16.8 ± 5.9	18.4 ± 5.9	21.2 ± 10.5	19.4 ± 6.4	0.004	0.01
C28:0	39.3 ± 20.7	32.3 ± 17.3	35.5 ± 15.9	39.9 ± 19.2	46.4 ± 26.1	38.6 ± 13.5	0.004	0.006
C30:1	10.1 ± 4.4	8.6 ± 3.7	9.2 ± 3.5	10.0 ± 3.9	11.7 ± 5.6	10.4 ± 2.8	0.002	0.005
Total SM	87,445.8 ± 23,733.7	79,737.7 ± 17,568.1	91,887.4 ± 26,135.4	90,567.5 ± 23,851.7	88,174.6 ± 25,995.3	81,765.5 ± 16,395.2	0.07	0.04
Total VLC SM	82.5 ± 36.2	70.9 ± 31.2	75.88 ± 27.7	82.1 ± 32.0	95.3 ± 46.3	81.8 ± 25.1	0.005	0.007
C18/C18:1	1.3 ± 0.2	1.3 ± 0.2	1.2 ± 0.2	1.2 ± 0.2	1.3 ± 0.2	1.3 ± 0.2	0.04	0.03
So	121.1 ± 54.8	157.6 ± 43.0	122.5 ± 50.7	106.2 ± 54.3	113.4 ± 54.6	100.9 ± 57.5	< 0.0001	< 0.0001
S1P	412.5 ± 187.1	401.6 ± 179.7	439.1 ± 194.1	354.7 ± 139.5	448.5 ± 216.7	440.0 ± 188.1	0.03	0.02

n = 240; data are shown as means ± SD; *SPL* sphingolipid, *Cer* ceramide, *MHC* monohexosylceramide, *LacCer* lactosylceramide, *SM* sphingomyelin, *VLC* very long chain. ^a^Unadjusted *p* value. ^b^*p* value adjusted for BMI, FPG, sex, and ethnicity

Among the MHC species, we observed significant differences between five MHC species across different age groups (C18:1, C26:0, C28:0, C34:1, C34:0) (Table 2) and after adjusting for BMI, FPG, sex, and ethnicity, three of them remained significant, MHC C18:1 (p = 0.02), C28:0 (p = 0.0009), and C34:0 (p = 0.009) (Table 2). We observed a trend of decreasing levels of very-long-chain MHC species with aging (for instance, the level of MHC C28:0 dropped by almost 59% from 2.2 ± 1.7 pmol/ml in the 19–29 y group to 0.9 ± 0.6 pmol/ml in the 60–65 y age group) (Table 2). We found that the ratios of saturated to monounsaturated for C18 and C26 species of MHC were significantly different across the age groups (Table 2).

Out of eighteen LacCer species, we found significant alteration of six species across different age groups (C26:1, C28:1, C30:1, C32:1, C34:1, C34:0), even after adjusting for BMI, FPG, sex, and ethnicity (Table 2). We observed a pattern of declining levels of certain very-long-chain LacCer species (C26:1 and C32:0) with aging, whereas some (C28:1, C30:1, C34:0) showed an increasing tendency with aging (Table 2). We also found that the ratio of saturated to monounsaturated LacCer C26 species significantly differed across the age groups (Table 2).

On the other hand, we observed that the plasma levels of six out of eighteen SM species were significantly altered across different age groups (C14:0, C18:1, C26:0, C28:1, C28:0, C30:1) and remained significant even after adjusting for BMI, FPG, sex, and ethnicity (Table 2). We witnessed a pattern of increasing levels of short-, long- and very-long-chain SM species with aging. For instance, the levels of SM C28:1 and C28:0 increased from 15.8 ± 8.1 and 32.3 ± 17.3 pmol/ml in the relatively younger group (19–29) y to 21.2 ± 10.5 and 46.4 ± 26.1 pmol/ml in the older one (50–59 y), respectively (Table 2). Moreover, the levels of total SM and total VLC SM also increased with aging (Table 2). We found that the ratio of saturated to monounsaturated SM C18 species significantly differed across the age groups (Table 2).

Analysis of 76 sphingolipid species after Bonferroni correction for multiple comparisons, with a significance threshold set at p < 0.0006, revealed significant associations between age and the ratio of Cer C18:0/C18:1, MHC C28:0, the MHC ratio of C26:0/C26:1, LacCer C32:0, LacCer C34:1, LacCer C34:0 (Table 2), and sphingosine (157.6 ± 43.0 pmol/ml in the younger group [19–29 y] to 109.9 ± 57.5 pmol/ml in the relatively older one [60–65 y]; P < 0.0001)(Fig. 1C). Among these, LacCer C32:0, LacCer C34:0, and sphingosine (So) remained significantly associated with age after adjusting for BMI, FPG, sex, and ethnicity with P < 0.0001(Table 2).

### Correlation of plasma SPL profile with age

**Fig. 3:**
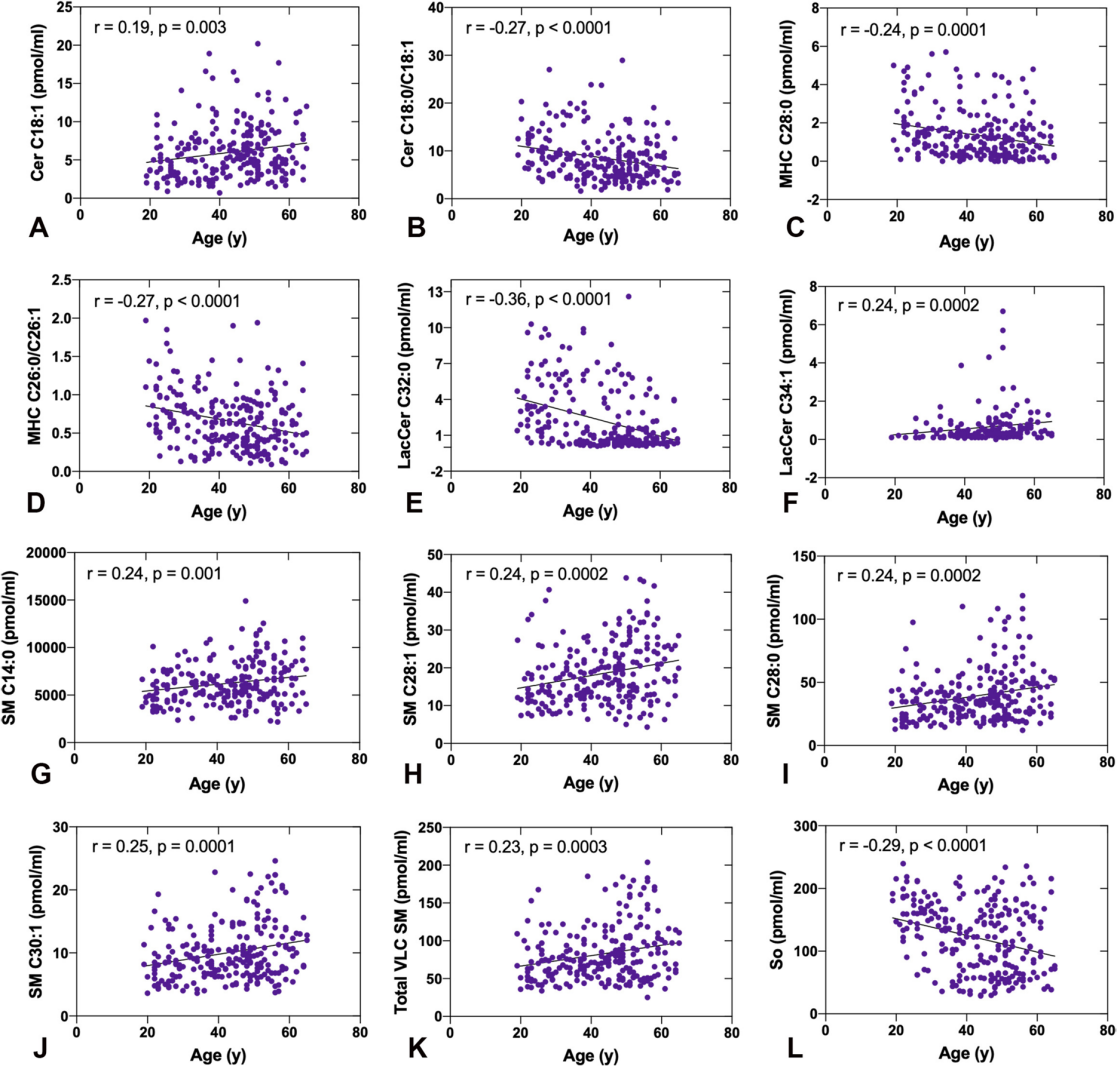
Certain species of ceramide, monohexosylceramide, lactosylceramide, sphingomyelin, and sphingosine showing strong association with age. **A** Cer C18:1, **B** Cer C18:0/C18:1, **C** MHC C28:0, **D** MHC C26:0/C26:1, **E** LacCer C32:0, **F** LacCer C34:1, **G** SM C14:0, **H** SM C28:1, **I** SM C28:0, **J** SM C30:1, **K** Total VLC SM, and **L** Sphingosine

SPL species variability in age-segregated populations showed species that are significantly different at different ages in the plasma samples of a normoglycemic population. We wanted to check if they correlate with age as a continuous variable. First, we checked those species and ratios that were significantly altered even after correction for multiple comparisons (Table 2). Although the changes in Cer C18:1 across age groups were no longer statistically significant after correction for multiple comparisons, we highlight this data due to its robust positive correlation with age (r = 0.19, p < 0.01) (Fig. 3A).

Accordingly, the Cer C18:0/C18:1 ratio (r = − 0.27, p < 0.0001) showed a very significant negative correlation with aging (Fig. 3B). In addition, very long chain MHC C28:0 showed very significant negative association with aging (r = − 0.24, p < 0.001) (Fig. 3C). MHC ratio C26:0/C26:1 (r = − 0.27, p < 0.0001) also followed suit, showing the strongest negative association with aging (Fig. 3D). Lactosylceramide species C32:0 showed significant negative association with aging, while C34:1 showed positive correlations with aging (Figs. 3E, F). In contrast to other sphingolipids, the correlation between sphingomyelin and aging generally exhibited a positive trend, with the exception of very long-chain sphingomyelins (VLCs) SM C34:1 and C34:0, which showed a negative association with age. For example, the short-chain SM C14:0 (r = 0.24, p = 0.001), as well as the very long-chain SM species C28:1, C28:0 (r = 0.24, p = 0.0002), C30:1 (r = 0.25, p = 0.0001), and total VLC SM (r = 0.25, p = 0.0001), all demonstrated strong positive correlations with aging (Figs. 3G–K). Plasma levels of So also maintained a very strong negative correlation with aging (Fig. 3L).

Besides those species described in Fig. 3, we also found several other SPL species correlated variably with aging. As presented in Table 3, Cer species, C16:0, showed a significant negative association with age, plasma very long chain MHC C26:0, C32:0, and C34:0 species showed very significant negative association with aging (Table 3), as well as MHC ratios C18:0/C18:1 (r = − 0.23, p = 0.0004) (Table 3).

**Table 3:** Correlation of ceramide, monohexosylceramide, lactosylceramide, sphingomyelin and sphingosine with age

Cer	r	MHC	r	LacCer	r	SM	r
C14:0	0.06	C14:0	0.02	C14:0	0.09	C14:0	0.20^**^
C16:0	− 0.13^*^	C16:0	− 0.11	C16:0	− 0.14^*^	C16:0	− 0.008
C18:1	0.20^**^	C18:1	0.11	C18:1	0.01	C18:1	0.05
C18:0	0.20	C18:0	− 0.07	C18:0	− 0.03	C18:0	0.06
C20:0	0.01	C20:0	− 0.12	C20:0	− 0.07	C20:0	0.08
C22:0	− 0.06	C22:0	− 0.12	C22:0	− 0.07	C22:0	0.05
C24:1	− 0.02	C24:1	− 0.12	C24:1	− 0.16^*^	C24:1	0.09
C24:0	− 0.09	C24:0	− 0.13^*^	C24:0	− 0.15^*^	C24:0	0.06
C26:1	0.04	C26:1	0.04	C26:1	− 0.17^**^	C26:1	0.12
C26:0	− 0.06	C26:0	− 0.20^**^	C26:0	− 0.16^*^	C26:0	0.07
C28:1	− 0.01	C28:1	− 0.11	C28:1	0.16^*^	C28:1	0.24^***^
C28:0	0.08	C28:0	− 0.24^***^	C28:0	− 0.14^*^	C28:0	0.24^***^
C30:1	0.07	C30:1	− 0.004	C30:1	0.23	C30:1	0.25^***^
C30:0	− 0.07	C30:0	− 0.09	C30:1	0.23^***^	C30:0	0.14^*^
C32:1	0.04	C32:1	− 0.05	C32:1	0.11	C32:1	0.07
C32:0	− 0.03	C32:0	− 0.20^**^	C32:0	− 0.36^****^	C32:0	− 0.03
C34:1	0.03	C34:1	− 0.10	C34:1	0.24^***^	C34:1	− 0.15^*^
C34:0	− 0.02	C34:0	− 0.22^***^	C34:0	0.31^****^	C34:0	− 0.16^*^
Total Cer	− 0.08	Total MHC	− 0.13^*^	Total LacCer	− 0.16^*^	Total SM	0.07
Total VLC Cer	0.04	Total VLC− MHC	− 0.14^*^	Total VLC LacCer	− 0.06	Total VLC SM	0.23^***^
C16:0/C18:0	− 0.05	C16:0/C18:0	0.05	C16:0/C18:0	0.05	C16:0/C18:0	− 0.08
C18/C18:1	− 0.27^****^	C18/C18:1	− 0.23^***^	C18/C18:1	0.03	C18/C18:1	0.03
C24:0/C24:1	− 0.08	C24:0/C24:1	− 0.02	C24:0/C24:1	0.05	C24:0/C24:1	− 0.01
C26:0/C26:1	− 0.08	C26/C26:1	− 0.27^****^	C26:0/C26:1	0.11	C26:0/C26:1	− 0.03

n = 240, values in table are for Pearson correlation coefficient (r). *Cer* ceramide, *MHC* monohexosylceramide, *LacCer* lactosylceramide, *SM* sphingomyelin, Significance values: Not significant (…) for ^*^p < 0.05, ^**^p < 0.01, ^***^p < 0.001, ^****^p < 0.0001

Lactosylceramide species largely followed the same trend as MHCs, showing a negative association with age. For instance, we observed that some VLC LacCer species showed a significant negative association with aging, except for LacCer C28:1, C30:1, C34:1, and C34:0, which showed positive correlations with aging (Table 3).

Unlike other sphingolipids, the correlation of sphingomyelin with aging largely followed a positive trajectory, apart from VLCs SM C34:1 and C34:0, which showed a negative association with age (Table 3). Our analysis also identified several sphingolipid species across different classes that showed no significant age-related changes (Table 3). These findings suggest that while certain sphingolipid species exhibit clear age-dependent variations, others maintain a stable profile throughout the age range we studied, implying that these species may play consistent roles in cellular function and homeostasis during aging.

## Discussion

Plasma lipidome profile has increasingly been investigated in many human health and diseases due to their potential to serve as important biomarkers for diagnosis and prognosis and as a valuable tool during therapeutic interventions. Plasma lipidome, which may consist of lipids of glycerolipids, phospholipids, fatty acids, glycolipids, and sphingolipids categories, represents more than 80% of the > 4,000 compounds identified with confirmation in plasma metabolome. In a homeostatic system, through a bidirectional interaction, the plasma profile of lipids may represent a state of cellular needs and specific physiological cell-tissue distribution. Consequently, the physiological state of cell-tissue-organ in aging, in diseases, or in pre-disease states can modify the chemical composition of blood plasma (Jove et al. 2016), supporting the association of specific lipidomic signatures with complex diseases such as Alzheimer’s disease, cardiovascular disease, and metabolic disorders (Dagogo-Jack et al. 2024; Hinterwirth et al. 2014; Mandal et al. 2024; Naudi et al. 2015). In addition to lipid roles related to membrane structure and energy metabolism, in the past years, sphingolipids have been conceived as information-carrying molecules because of their implication in signaling processes, including cell-stress responses, cell survival, or inflammation (Hannun and Obeid 2018; Mandal et al. 2021). In this study, we investigated the relationship between 76 species of plasma sphingolipids, their levels, and the ratios of saturated/monounsaturated species with aging in a cohort of normal, healthy 240 black and white participants aged between 19 and 65 years. We found significant age-related changes in several sphingolipid species: (1) ceramide C18:1 and several long-chain sphingomyelins (VLC SMs), such as C28:1 and C30:1, were positively correlated with age; (2) glycosphingolipids (MHC and LacCer) showed strong negative correlations with aging; (3) sphingosine levels also showed significant strong negative correlation with aging; and (4) the ratios of saturated/monounsaturated species were negatively associated with age, indicating the accumulation of monounsaturated species in the plasma with aging.

Sphingolipids are highly conserved cell membrane lipids crucial for forming important membrane domains, the lipid rafts. The metabolism of sphingolipids is very complex in which ceramide sits in a central position, which can be synthesized by three main pathways: the de novo pathway, hydrolysis of complex sphingolipids, and the salvage pathway (Hannun and Obeid 2018; Mandal et al. 2021; Paranjpe et al. 2022; Simon et al. 2021). Structural sphingolipids include sphingomyelins and glycosphingolipids (including HexCer, LacCer, gangliosides (GM), and sulfatides), whereas bioactive sphingolipids are represented by sphingosine, S1P, ceramide 1-phosphate (C1P), dihydroceramide (dhCer), and ceramides (Hannun and Obeid 2018; Mandal et al. 2021; Paranjpe et al. 2022; Simon et al. 2021). Functionally, SPLs are involved in regulating membrane physiology and related functions such as membrane fluidity, organization, geometry, lipid-protein and protein–protein interactions, membrane trafficking, clustering of plasma membrane receptors and ion channels, signal transduction, as well as cytoskeletal organization (Hannun and Obeid 2018; Mandal et al. 2021; Paranjpe et al. 2022; Simon et al. 2021). At the cellular levels, SPLs signal for oxidative stress, apoptosis, cell survival, autophagy, endosomes and endocytosis, cell cycle, proliferation, migration, and senescence and at complex physiological levels, SPLs are involved in development, aging, and life span and in several pathological conditions such as inflammation, metabolic and cardiovascular diseases, neurodegeneration, and cancer (Hannun and Obeid 2018; Mandal et al. 2021; Paranjpe et al. 2022; Simon et al. 2021).

Despite the well-known presence and importance of sphingolipids in living organisms and their significant impact on human health, comprehensive studies focusing on the detection, quantification, and aging-related changes in sphingolipid profiles in plasma or in tissue, particularly with large cohorts, remain limited. Emerging evidence suggests ceramide accumulation plays a critical role in the aging process and in age-related diseases (Czubowicz et al. 2019; Shu et al. 2022). Ceramides, bioactive lipids involved in multiple cellular processes such as inflammation, stress response, insulin signaling, and apoptosis, are thought to contribute to pro-inflammatory and lipotoxic states that are characteristic of aging (Stith et al. 2019). We found total ceramides in plasma did not change significantly between the ages 20 to 65 years except for one species, Cer C18:1, which positively correlated with age (Fig. 3; Tables 2 and 3). We also found significant alteration of C18:1 or monounsaturated oleic acid-containing species in the MHC and SM groups (Table 2); however, C18:1 MHC or SM do not correlate with age (Table 3). C18:1 also serves as a precursor for longer-chain monounsaturated fatty acids (MUFA) such as C24:1 and C26:1 and higher. We found a significant correlation of saturate/ monounsaturated C18 Cer, MHC, and C26 MHC with age (Table 3). Oleic acid is synthesized from the most abundant saturated fatty acid in our diet, stearic acid (C18:0) by the enzyme Sreroyl CoA desaturase (SCD1)(Loix et al. 2024). SCD1 genotype variants are associated with human metabolic diseases, and increased activity of this enzyme has been found in many forms of age-related diseases (Hulver et al. 2005; Loix et al. 2024; Mar-Heyming et al. 2008; Paton and Ntambi 2009; Rahman et al. 2003). Inhibition of SCD1 in many disease models showed beneficial effects; however, complete inhibition or knocking out causes mucolipodystrophy, skin inflammation in humans, and other adverse consequences in model organisms (Liu et al. 2011; Loix et al. 2024). Therefore, a balanced expression of this protein and its activity is important for healthy living and healthy aging. Previous studies in the same cohort identified higher levels of MUFA in the plasma in individuals significantly associated with a family history of diabetes (Mandal et al. 2024); and very interestingly, among the healthy participants who progressed to prediabetes in the next 5 year-follow-up had significantly higher saturated/monounsaturated ratios (lower levels of MUFA) of ceramides C18:0/C18:1 and C26:0/C26:1, and sphingomyelins C18:0/C18:1, C24:0/C24:1, and C26:0/C26:1, in baseline plasma specimens (Dagogo-Jack et al. 2024). MUFAs, particularly oleic acid (C18:1), are increasingly recognized for their anti-inflammatory and metabolic regulatory roles that support healthy aging (Lopez-Huertas 2010; Pararasa et al. 2016; Stromsnes et al. 2021). Replacing saturated fats with MUFAs has been shown to improve lipid profiles, reduce cardiovascular risk, and enhance insulin sensitivity (Lopez-Huertas 2010). Mechanistically, MUFAs promote an anti-inflammatory immune profile by driving macrophage polarization toward a pro-resolving M2 phenotype via PPARγ activation and oxidative phosphorylation, whereas saturated fatty acids induce a pro-inflammatory M1 phenotype through ceramide accumulation and NF-κB signaling (Pararasa et al. 2016). Notably, inflammation-related changes in circulating fatty acid composition are observed with age, where elevated levels of saturated fatty acids correlate with higher pro-inflammatory cytokines (TNF-α, IL-6) and lower anti-inflammatory mediators (IL-10), contributing to the chronic inflammatory state known as inflammaging (Pararasa et al. 2016). In our study, we observed consistent age-associated decreases in the ratio of saturated to monounsaturated sphingolipid species, specifically Cer C18:0/C18:1 and MHC C26:0/C26:1, suggesting a metabolic shift toward MUFA-enriched lipid pools in older individuals. This shift may reflect an adaptive lipidomic remodeling aimed at preserving membrane fluidity, signaling integrity, and cellular resilience under inflammatory stress (Stromsnes et al. 2021). Furthermore, endogenous MUFA production via stearoyl-CoA desaturase-1 (SCD1) has been shown to protect against hepatic steatosis and insulin resistance, reinforcing the view that MUFA enrichment plays a compensatory and protective role in age-related metabolic regulation (Sampath and Ntambi 2011). These findings suggest that MUFAs may serve as crucial modulators of systemic inflammation in aging, offering a promising dietary and metabolic strategy to mitigate age-related inflammatory decline and promote healthier longevity. Taken together, our observations further emphasize the fact that a balanced level of saturated/monounsaturated species SPLs is very important for health, and an increase of MUFA in a healthy aging population may indicate their roles in metabolic shift toward cellular senescence, inflammation, and lipotoxicity, hallmark features of aging and underscoring the role of sphingolipid metabolism in aging.

We observed plasma short-chain SM, C14:0 increases with aging (Table 3), and we also found significant positive correlations of very-long-chain sphingomyelins (VLC SMs: C28-C32) with aging; the group of SPL species has not been investigated in any previous studies. These findings are in agreement with earlier reports that have connected sphingomyelins to age-related processes (Mielke et al. 2015). Previous studies reported an increase in plasma SM levels with age (Mielke et al. 2015) also a direct association between elevated plasma SM and risk of Alzheimer’s disease, a neurodegenerative condition closely linked to aging (Mielke et al. 2017). Notably, sphingomyelin levels are not only elevated in plasma but also in tissues, as significant age-related increases are observed in the hippocampus demonstrating a strong correlation between plasma and tissue content of SM (Couttas et al. 2018). Acid sphingomyelinase (ASM), an enzyme that hydrolyzes SM to ceramides, has been speculated to have a role in maintaining plasma SM levels and may play important roles in SM, ceramide ratio in plasma and has been investigated as a target for novel therapies for age-associated diseases (Henry et al. 2013; Park et al. 2020, 2018). The specific roles of VLC SM in the aging population, whether they are protective or harmful, however, need further investigation.

In contrast to ceramides and SM, the levels of glycosphingolipids, such as MHCs and LacCers, exhibited strong inverse associations with aging (Table 3). Very-long-chain MHC species, including C26:0, C28:0, and C34:0, along with the C18:0/C18:1 ratio, were negatively correlated with aging (Table 3). These findings suggest that aging is accompanied by a decline in glycosphingolipid metabolism, particularly the reduced levels of VLC MHC species. VLC SPL species play critical roles in maintaining membrane integrity, cell signaling, and lipid raft formation (Iwabuchi 2015; Naudi et al. 2013; Skowronska-Krawczyk and Budin 2020), and their reduction with age could indicate compromised cellular communication and membrane stability, key features of age-related diseases such as neurodegeneration and cardiovascular disorders (Czubowicz et al. 2019; Shu et al. 2022). Similarly, we observed that the levels of VLC LacCers (C26:1, C32:0) were higher in the younger participants compared to the older groups (Table 2). Elevated glycosphingolipids have been linked to healthy aging and longevity, as seen in centenarians who exhibit higher levels of these lipids (Pradas et al. 2022). As they participate in lipid raft formation, disruptions in lipid raft composition are linked to aging and various age-related diseases (Trayssac et al. 2018). Healthy, functional membranes are crucial for cellular resilience to stress, potentially contributing to the maintenance of healthy aging (Trayssac et al. 2018). Thus, the higher VLC MHC and LacCer levels in younger individuals could reflect a protective feature that aids in cellular integrity and longevity.

Sphingosine (So) levels in plasma decreased with age and showed a robust negative correlation with aging (Figs. 1 and 3). So is a long-chain base containing amino alcohol, and simple SPL but has a signaling role. So signaling matches mostly with ceramides as they signal for apoptosis and senescence (Bartke and Hannun 2009; Simon et al. 2021); it also exerts an antagonistic effect on calcium-dependent protein kinases, which are involved in various aging-related cellular processes (Engin and Engin 2021). So is generated mainly by the hydrolysis of complex SPL in lysosome by lysosomal acidic enzymes such as ASM and acid Ceramidase and serves as the sole precursor for another important bioactive SPL, S1P (Bartke and Hannun 2009; Paranjpe et al. 2022; Simon et al. 2021). S1P is considered to be a beneficial signaling molecule that works through five known G-protein coupled receptors (S1PR1-5) and affects multiple cellular functions that include cell proliferation, adhesion, migration, inflammation, immunomodulation and also a potent cellular antagonist for ceramide lipotoxicity (Maceyka et al. 2012; Maceyka and Spiegel 2014; Spiegel 2020). Our observation of SM changes and So decreases with age may have significant implications for understanding how SPL homeostasis shifts as we age. It also indicates a reduced lysosomal activity may be responsible for this trend. Lysomosal activity is very important for cellular autophagy, and declining lysosomal activity and autophagy are associated with all forms of age-associated diseases (Aman et al. 2021; Tan and Finkel 2023). Further investigation of SM and So plasma levels and their validation may generate novel biomarkers for healthy aging and age-associated diseases.

Our findings support the notion that certain sphingolipid profiles may be linked to longevity. Age-related alterations in sphingolipid profiles have been studied in experimental model organisms, revealing several key changes that coincide with aging. For instance, in yeast, *Saccharomyces cerevisiae*, sphingolipid biosynthesis inhibition negatively impacts the activity of sphingolipid-regulated Pkh1/2 protein kinases and one of their downstream targets, the Sch9 protein kinase, resulting in an increased chronological life span of the animal (Huang et al. 2012). In a study involving the worm *Caenorhabditis elegans*, pharmacological inhibitors and small interfering RNAs directed against Serine palmitoyl transferase and Glucosylceramide synthase acted to slow the development rate, extend the reproductive period and increase the lifespan of the organism (Cutler et al. 2014). In a study with a long-lived naked molerat (*Heterocephalus glaber*) known as a mammal with negligible senescence, an association has been found between sphingolipid content and animal longevity (Lewis et al. 2018), providing some evidence of the roles of sphingolipid metabolism in regulating development and lifespan. In human studies, profiling plasma sphingolipids in centenarians, Pradas and colleagues (Pradas et al. 2022) identified sphingolipid signatures associated with longevity, lower concentrations of ceramide biosynthesis intermediates, sphingomyelins, and sulfatides, and higher levels of hexosylceramides and gangliosides. This signature closely resembles the sphingolipid profile observed in our younger cohort, with lower levels of ceramides and sphingomyelins and higher levels of glycosphingolipids. This convergence between the profiles of centenarians and our younger participants suggests that maintaining a youthful sphingolipid profile may play a role in healthy aging and extended lifespan. Other studies have shown that centenarians exhibit resistance to typical age-related lipid changes, maintaining a more youthful lipidome (Jove et al. 2017). Additionally, Montoliu et al. (Montoliu et al. 2014) hypothesized that centenarians, from a metabolic perspective, are biologically younger than their chronological age, further supporting the idea that maintaining lipid homeostasis could be crucial for longevity. As dysfunctional telomeres are associated with senescence (Lendvay et al. 1996), a protective effect of sphingolipids on telomere homeostasis may be a unifying mechanism for the association of sphingolipid signatures with longevity (Ikeda et al. 2015)**.**

Our study provides valuable insights into the sphingolipid-age association in a large cohort of biracial participants, but there are limitations. The targeted lipidomics approach used in our study may have missed potentially significant yet unknown lipids. Additionally, the lack of full ethnic diversity is an important limitation. Future studies should employ a broader lipidomic profiling approach, combining targeted and shotgun lipidomics in an even larger and more ethnically diverse cohort that spans a broader age range.

In conclusion, our study provides important insights into the plasma distribution of sphingolipids across different age groups, revealing distinct patterns of sphingolipid metabolism with aging. We identified two main trends: ceramides (e.g., Cer C18:1) and sphingomyelins (e.g., C28:1, C28:0, C30:1) were upregulated in older participants, while glycosphingolipids (e.g., MHC, LacCer) and sphingosine were downregulated, with younger individuals exhibiting higher levels of these species. These findings suggest that ceramides and sphingomyelins may contribute to the aging process, while glycosphingolipids and sphingosine may help maintain cellular function and resilience in younger individuals. Importantly, maintaining a youthful sphingolipid profile, characterized by higher levels of glycosphingolipids and sphingosine, may be crucial for promoting healthy aging and longevity. Our results offer potential avenues for future research aimed at modulating sphingolipid metabolism to support healthy aging.

## Supplementary Information

Below is the link to the electronic supplementary material.

Supplementary file1 (DOCX 24 KB)

## Data Availability

All data that support the findings of this study are available from the corresponding author upon reasonable request.
