# Supplementary material for: Do circulating sphingolipid species correlate with age? A study in a normoglycemic biracial population
Source: Biogerontology. 2025 May 5;26(3):106. doi: 10.1007/s10522-025-10244-9 (PMC12052799; doi:10.1007/s10522-025-10244-9)
Supplement: Supplementary file 1 — Supplementary file1 (DOCX 24 KB) [file 10522_2025_10244_MOESM1_ESM.docx]

**SUPPLEMENTARY TABLE**

**Table S1.** LIPID MAPS nomenclature and the Sphingoid Base and N-acyl Fatty Acid composition of the Sphingolipid species analyzed by AB Sciex 5500 QTrap Mass Spectrometer.

| **Nomenclature**  **(This manuscript)** | **LIPID MAPS**  **(Molecular Species)** | **Sphingoid Base** | **N-Acyl Fatty acid** |
| --- | --- | --- | --- |
| ***Ceramides (Cer)*** |  |  |  |
| Cer C12:0 | Cer d18:1/12:0 | d18:1 | C12:0 |
| Cer C14:0 | Cer d18:1/14:0 | d18:1 | C14:0 |
| Cer C16:0 | Cer d18:1/16:0 | d18:1 | C16:0 |
| Cer C18:1 | Cer d18:1/18:1 | d18:1 | C18:1 |
| Cer C18:0 | Cer d18:1/18:0 | d18:1 | C18:0 |
| Cer C20:0 | Cer d18:1/20:0 | d18:1 | C20:0 |
| Cer C22:0 | Cer d18:1/22:0 | d18:1 | C22:0 |
| Cer C24:1 | Cer d18:1/24:1 | d18:1 | C24:1 |
| Cer C24:0 | Cer d18:1/24:0 | d18:1 | C24:0 |
| Cer C26:1 | Cer d18:1/26:1 | d18:1 | C26:1 |
| Cer C26:0 | Cer d18:1/26:0 | d18:1 | C26:0 |
| Cer C28:1 | Cer d18:1/28:1 | d18:1 | C28:1 |
| Cer C28:0 | Cer d18:1/28:0 | d18:1 | C28:0 |
| Cer C30:1 | Cer d18:1/30:1 | d18:1 | C30:1 |
| Cer C30:0 | Cer d18:1/30:0 | d18:1 | C30:0 |
| Cer C32:1 | Cer d18:1/32:1 | d18:1 | C32:1 |
| Cer C32:0 | Cer d18:1/32:0 | d18:1 | C32:0 |
| Cer C34:1 | Cer d18:1/34:1 | d18:1 | C34:1 |
| Cer C34:0 | Cer d18:1/34:0 | d18:1 | C34:0 |
| ***Monohexosylceramides (MHC or HexCer)*** |  |  |  |
| MHC C12:0 | HexCer d18:1/12:0 | d18:1 | C12:0 |
| MHC C14:0 | HexCer d18:1/14:0 | d18:1 | C14:0 |
| MHC C16:0 | HexCer d18:1/16:0 | d18:1 | C16:0 |
| MHC C18:1 | HexCer d18:1/18:1 | d18:1 | C18:1 |
| MHC C18:0 | HexCer d18:1/18:0 | d18:1 | C18:0 |
| MHC C20:0 | HexCer d18:1/20:0 | d18:1 | C20:0 |
| MHC C22:0 | HexCer d18:1/22:0 | d18:1 | C22:0 |
| MHC C24:1 | HexCer d18:1/24:1 | d18:1 | C24:1 |
| MHC C24:0 | HexCer d18:1/24:0 | d18:1 | C24:0 |
| MHC C26:1 | HexCer d18:1/26:1 | d18:1 | C26:1 |
| MHC C26:0 | HexCer d18:1/26:0 | d18:1 | C26:0 |
| MHC C28:1 | HexCer d18:1/28:1 | d18:1 | C28:1 |
| MHC C28:0 | HexCer d18:1/28:0 | d18:1 | C28:0 |
| MHC C30:1 | HexCer d18:1/30:1 | d18:1 | C30:1 |
| MHC C30:0 | HexCer d18:1/30:0 | d18:1 | C30:0 |
| MHC C32:1 | HexCer d18:1/32:1 | d18:1 | C32:1 |
| MHC C32:0 | HexCer d18:1/32:0 | d18:1 | C32:0 |
| MHC C34:1 | HexCer d18:1/34:1 | d18:1 | C34:1 |
| MHC C34:0 | HexCer d18:1/34:0 | d18:1 | C34:0 |
| ***Lactosylceramides (LacCer)*** |  |  |  |
| LacCer C12:0 | Cer d18:1/12:0 | d18:1 | C12:0 |
| LacCer C14:0 | LacCer d18:1/14:0 | d18:1 | C14:0 |
| LacCer C16:0 | LacCer d18:1/16:0 | d18:1 | C16:0 |
| LacCer C18:1 | LacCer d18:1/18:1 | d18:1 | C18:1 |
| LacCer C18:0 | LacCer d18:1/18:0 | d18:1 | C18:0 |
| LacCer C20:0 | LacCer d18:1/20:0 | d18:1 | C20:0 |
| LacCer C22:0 | LacCer d18:1/22:0 | d18:1 | C22:0 |
| LacCer C24:1 | LacCer d18:1/24:1 | d18:1 | C24:1 |
| LacCer C24:0 | LacCer d18:1/24:0 | d18:1 | C24:0 |
| LacCer C26:1 | LacCer d18:1/26:1 | d18:1 | C26:1 |
| LacCer C26:0 | LacCer d18:1/26:0 | d18:1 | C26:0 |
| LacCer C28:1 | LacCer d18:1/28:1 | d18:1 | C28:1 |
| LacCer C28:0 | LacCer d18:1/28:0 | d18:1 | C28:0 |
| LacCer C30:1 | LacCer d18:1/30:1 | d18:1 | C30:1 |
| LacCer C30:0 | LacCer d18:1/30:0 | d18:1 | C30:0 |
| LacCer C32:1 | LacCer d18:1/32:1 | d18:1 | C32:1 |
| LacCer C32:0 | LacCer d18:1/32:0 | d18:1 | C32:0 |
| LacCer C34:1 | LacCer d18:1/34:1 | d18:1 | C34:1 |
| LacCer C34:0 | LacCer d18:1/34:0 | d18:1 | C34:0 |
| ***Sphingomyelins (SM)*** |  |  |  |
| SM C12:0 | SM d18:1/12:0 | d18:1 | C12:0 |
| SM C14:0 | SM d18:1/14:0 | d18:1 | C14:0 |
| SM C16:0 | SM d18:1/16:0 | d18:1 | C16:0 |
| SM C18:1 | SM d18:1/18:1 | d18:1 | C18:1 |
| SM C18:0 | SM d18:1/18:0 | d18:1 | C18:0 |
| SM C20:0 | SM d18:1/20:0 | d18:1 | C20:0 |
| SM C22:0 | SM d18:1/22:0 | d18:1 | C22:0 |
| SM C24:1 | SM d18:1/24:1 | d18:1 | C24:1 |
| SM C24:0 | SM d18:1/24:0 | d18:1 | C24:0 |
| SM C26:1 | SM d18:1/26:1 | d18:1 | C26:1 |
| SM C26:0 | SM d18:1/26:0 | d18:1 | C26:0 |
| SM C28:1 | SM d18:1/28:1 | d18:1 | C28:1 |
| SM C28:0 | SM d18:1/28:0 | d18:1 | C28:0 |
| SM C30:1 | SM d18:1/30:1 | d18:1 | C30:1 |
| SM C30:0 | SM d18:1/30:0 | d18:1 | C30:0 |
| SM C32:1 | SM d18:1/32:1 | d18:1 | C32:1 |
| SM C32:0 | SM d18:1/32:0 | d18:1 | C32:0 |
| SM C34:1 | SM d18:1/34:1 | d18:1 | C34:1 |
| SM C34:0 | SM d18:1/34:0 | d18:1 | C34:0 |
| ***Sphingoid Bases (SPB)*** |  |  |  |
| Sphingosine (So) | SPB d18:1 | d18:1 |  |
| Dihydrosphingosine (DHSo) | SPB d18:0 | d18:0 |  |
| Sphingosine 1-phosphate (S1P) | SPBP d18:1 | d18:1 |  |
| Dihydrosphingosine 1-phosphate (DH-S1P) | SPBP d18:0 | d18:0 |  |
